# Remote Patient Monitoring and Incentives to Support Smoking Cessation Among Pregnant and Postpartum Medicaid Members: Three Randomized Controlled Pilot Studies

**DOI:** 10.2196/27801

**Published:** 2021-09-30

**Authors:** Caroline M Joyce, Kathryn Saulsgiver, Salini Mohanty, Chethan Bachireddy, Carin Molfetta, Mary Steffy, Alice Yoder, Alison M Buttenheim

**Affiliations:** 1 Department of Epidemiology Faculty of Medicine McGill University Montreal, QC Canada; 2 BetterUp San Francisco, CA United States; 3 Department of Family and Community Health School of Nursing University of Pennsylvania Philadelphia, PA United States; 4 School of Medicine Virginia Commonwealth University Richmond, VA United States; 5 Penn Medicine Lancaster General Health Lancaster, PA United States; 6 Center for Health Incentives and Behavioral Economics Perelman School of Medicine University of Pennsylvania Philadelphia, PA United States

**Keywords:** maternal smoking, smoking cessation, financial incentives, smoking, pregnant, postpartum, incentives, mHealth, mobile health, mobile phone, smart devices

## Abstract

**Background:**

Smoking rates among low-income individuals, including those eligible for Medicaid, have not shown the same decrease that is observed among high-income individuals. The rate of smoking among pregnant women enrolled in Medicaid is almost twice that among privately insured women, which leads to significant disparities in birth outcomes and a disproportionate cost burden placed on Medicaid. Several states have identified maternal smoking as a key target for improving birth outcomes and reducing health care expenditures; however, efficacious, cost-effective, and feasible cessation programs have been elusive.

**Objective:**

This study aims to examine the feasibility, acceptability, and effectiveness of a smartwatch-enabled, incentive-based smoking cessation program for Medicaid-eligible pregnant smokers.

**Methods:**

Pilot 1 included a randomized pilot study of smartwatch-enabled remote monitoring versus no remote monitoring for 12 weeks. Those in the intervention group also received the SmokeBeat program. Pilot 2 included a randomized pilot study of pay-to-wear versus pay-to-quit for 4 weeks. Those in a pay-to-wear program could earn daily incentives for wearing the smartwatch, whereas those in pay-to-quit program could earn daily incentives if they wore the smartwatch and abstained from smoking. Pilot 3, similar to pilot 2, had higher incentives and a duration of 3 weeks.

**Results:**

For pilot 1 (N=27), self-reported cigarettes per week among the intervention group declined by 15.1 (SD 27) cigarettes over the study; a similar reduction was observed in the control group with a decrease of 17.2 (SD 19) cigarettes. For pilot 2 (N=8), self-reported cigarettes per week among the pay-to-wear group decreased by 43 cigarettes (SD 12.6); a similar reduction was seen in the pay-to-quit group, with an average of 31 (SD 45.6) fewer cigarettes smoked per week. For pilot 3 (N=4), one participant in the pay-to-quit group abstained from smoking for the full study duration and received full incentives.

**Conclusions:**

Decreases in smoking were observed in both the control and intervention groups during all pilots. The use of the SmokeBeat program did not significantly improve cessation. The SmokeBeat program, remote cotinine testing, and remote delivery of financial incentives were considered feasible and acceptable. Implementation challenges remain for providing evidence-based cessation incentives to low-income pregnant smokers. The feasibility and acceptability of the SmokeBeat program were moderately high. Moreover, the feasibility and acceptability of remote cotinine testing and the remotely delivered contingent financial incentives were successful.

**Trial Registration:**

ClinicalTrials.gov NCT03209557; https://clinicaltrials.gov/ct2/show/NCT03209557.

## Introduction

### Background

Smoking during pregnancy is the most preventable cause of infant morbidity, mortality, and pregnancy-related complications and is a key driver of higher health care costs [1-3]. Although smoking prevalence in the United States has declined over the past few decades, smoking prevalence remains higher among low-income individuals than among medium- and high-income individuals because of social and structural factors [4]. Currently, the smoking rates among pregnant women enrolled in Medicaid are almost twice the rate as that of privately insured women, leading to significant disparities in birth outcomes and a disproportionate cost burden placed on Medicaid [5,6]. Several states have identified maternal smoking as a key target for improving birth outcomes and reducing health care expenditures [7,8]; however, efficacious, cost-effective, and feasible cessation programs have been elusive.

Financial incentives for cessation have been shown repeatedly to reduce the occurrence of smoking in pregnancy [9-11], but few state Medicaid programs, payers, and health systems have scaled them up in practice. Previous incentive-based studies targeting pregnant women suffer from several drawbacks. First, they rely on an intensive in-person visit schedule that limits implementation at scale, which is a particular barrier for rural patient populations and those facing transportation challenges. Second, they have not successfully operationalized the frequent feedback and reward schedules that maximize effectiveness [12,13]. Third, few programs have developed valid and acceptable protocols for the remote, frequent biochemical verification of smoking abstinence (eg, cotinine testing and exhaled carbon monoxide sensing).

New technologies have recently expanded options available to support smoking cessation, including smartphone apps and remote patient monitoring (RPM) technologies to support smoking cessation. A recent entrant in this space is the SmokeBeat program (Somatix). When paired with a smartwatch or smartband worn on the wrist, the SmokeBeat program automatically detects cigarettes smoked through arm and wrist motion data generated by the wearable smartwatch or smartband. After a brief learning period, the smartwatch with good reliability can automatically detect cigarette use. A corresponding smartphone app provides a dashboard where participants can see their daily number of cigarettes smoked, average daily smoking, and other insights useful for cessation support. The app also allows targeted messages to be communicated to the smoker, which can influence smoking behavior. For example, participants enter the cost of a pack of cigarettes when they first set up the app, and the dashboard then shows the amount spent on cigarettes smoked on a given day, or the amount saved by abstaining. Previous research has shown the efficacy and reliability of SmokeBeat, and other similar programs, in detecting smoking and serving as a cessation aid [14-17]. These tools may be particularly helpful for low-income pregnant women who face scarcity in terms of time, resources, and cognitive bandwidth [18] but who are also in a unique window of opportunity and motivation to quit smoking to benefit their baby’s health as well as their own [10,19].

### Objectives

In this series of three small-scale, rapid cycle, randomized controlled pilot studies, we had four feasibility and acceptability goals and one effectiveness goal. We aim to assess the feasibility and acceptability by performing following tasks: (1) recruiting Medicaid-eligible pregnant smokers to participate in a smoking cessation study; (2) using the Somatix SmokeBeat program to remotely track participants’ smoking and provide feedback to participants and researchers; (3) conducting remote cotinine testing with study participants via video chat for the biochemical verification of smoking status; and (4) delivering incentives of different magnitudes contingent on smoking cessation or engagement with remote tracking technology. We also sought to preliminarily assess the effectiveness of the SmokeBeat program, with and without financial incentives, as a smoking cessation support tool for Medicaid-eligible pregnant smokers.

## Methods

### Setting

We conducted the rapid cycle pilot studies with pregnant and postpartum Medicaid members who were recruited through two pregnancy support programs (Healthy Beginnings Plus and Nurse Family Partnership) offered by Penn Medicine Lancaster General Health in Lancaster, Pennsylvania from 2017 to 2019.

### Participants

Women were eligible to participate if they were pregnant or recently postpartum, had an Android phone, were current smokers, and were currently participating in one of the two pregnancy support programs that serve Medicaid-eligible patients at Penn Medicine Lancaster General Health. Smoking was identified on the intake of these pregnancy support programs. As smoking cessation is not a requirement to be enrolled in these pregnancy support programs, participants did not have to endorse a desire to quit smoking to be counted as eligible for this research study. Eligible participants were identified by program staff and approached during a program visit or contacted by a text message to assess interest in joining a smoking cessation program. If interest was confirmed, then contact information was sent to the study coordinator at the research site. Study enrollment occurred over a phone call with a study coordinator who then sent a web-based consent form. Owing to limited sample pool in these hospital-based pregnancy support programs, all eligible participants were approached by study staff. Study supplies (smartwatch or smartband and charger, cotinine testing supplies, a ClinCard [a Health Insurance Portability and Accountability Act–compliant reloadable debit card for study payments], and mailing supplies) were either delivered via local program staff or mailed directly to the participants’ homes.

### Contact With Participants

Once enrolled, participants completed weekly video chats with a study coordinator via the Zoom (Zoom Video Communications) platform. During study visits, participants completed biological cotinine verification (saliva or urine samples), responded to a questionnaire, and received payment on their reloadable debit card. The questionnaire comprised six questions and included questions about self-rated stress over the last week, along with self-reported cigarette use (Multimedia Appendix 1). The study coordinator also checked how the smartwatch and app were functioning and helped with any technical issues encountered. The majority of these check-ins were <10 minutes. Participants were able to text or call the study coordinator with any questions or technical problems encountered during the course of their enrollment.

### Interventions

The intervention details for each pilot are listed in Table 1. Briefly, randomized participants (n=27) in pilot 1 received a smartwatch and assistance activating the SmokeBeat app and linking it to the smartwatch or to a control condition with no intervention. The participants were followed up for 12 weeks. All pilot 2 participants (n=8), a subsample of pilot 1 participants, received a smartwatch with the SmokeBeat app, and were randomized to a pay-to-wear condition (incentives earned were contingent on wearing the watch for 16 hours per day) or a pay-to-quit condition (incentives earned were contingent on wearing the watch and not recording any smoking events). Pilot 2 lasted 4 weeks. Pilot 3 (n=4) recruited a new sample group that was randomized to similar pay-to-wear versus pay-to-quit conditions as in pilot 2 but with higher incentive amounts and a proprietary Somatix wristband instead of a smartwatch. Incentive amounts for pilots 1 and 2 were selected based on the study budget and feasibility and sustainability of future scaled-up programs, though still in line with previous smoking cessation research [20]. The higher incentive amounts in pilot 3 were identified as being in line with previous smoking cessation incentives studies [10], including incentives studies with pregnant Medicaid members [21]. In all three pilots, all participants met weekly with the study staff for a check-in regardless of which condition they were randomized to, and were paid for these visits, following the research participation payment norms in this setting. All participants were also paid for intake and exit questionnaires. Incentive payments were processed daily after checking for the previous day’s smoking activity.

**Table 1:** Sample, intervention, and incentives details for the SmokeBeat pilots.

Pilot	Sample	Randomization	Duration	Intervention including cessation or engagement incentives	Remote video check-ins and cotinine testing	Participation incentives
1	A total of 27 (of 106 approached) pregnant smokers enrolled in Medicaid programs who had an Android smartphone.	A 2:1 randomization was used giving a ratio of 18 intervention: 9 controls.	12 weeks	Intervention: received smartwatch and SmokeBeat program, instructed on how to use program Control: no watch or program	Weekly video check-ins with study staff. Urine cotinine testing at weeks 1,4,7, 10; saliva cotinine test at weeks 2, 3, 5, 6, 8, 9, 11, 12	In total, US $25 for the completion of questionnaire at beginning and end of program+US $15 per weekly visit (12 total)+US $20 for qualitative interview at end of study=US $250 total
2	A total of 8, including 6 (of the original 27) Pilot 1 participants enrolled.	A 1:1 randomization was used giving a ratio of 4 intervention: 4 control.	4 weeks	Pay-to-wear: received smartwatch and SmokeBeat program. Eligible to receive US $1/day for wearing watch ≥16 hours per day=US $28 total Pay-to-quit: received smartwatch and SmokeBeat program. Eligible to receive incentives for wearing watch ≥16 hours per day and not recording any smoking events during that time. Streak-based incentives increased from US $1 per day up to US $7 per day, with reset if conditions not met or each week=US $112 total	Weekly video check-ins with study staff. Saliva cotinine testing every week	US $10 for 5 video check-in calls (consent+weekly study call)=US $50 total
3	A total of 4 participants (of 23 approached) eligible pregnant smokers enrolled in Medicaid programs who had an Android smartphone.	A 1:1 randomization was used giving a ratio of 2 intervention: 2 control.	3 weeks	Pay-to-wear: received smartwatch and SmokeBeat program. Eligible to receive US $3/day for wearing watch ≥16 hours per day plus US $50 bonus for wearing watch on 17/21 days=US $113 total Pay-to-quit: received smartwatch and SmokeBeat program. Eligible to receive incentives for wearing watch ≥16 hours per day and not recording any smoking events during that time. Streak-based incentives increased from US $5 per day by US $2 per day with reset if conditions not met or each week; US $50 bonus for not smoking for days 1 through 5 of the study; US $75 bonus for negative cotinine test at week 3=US $525 total	Weekly video check-ins with study staff. Saliva cotinine testing every week	US $10 for 4 video check-in calls (consent+weekly study call)=US $50 total

### Data Collection Procedures

#### Questionnaires

The participants completed questionnaires during weekly remote study visits. Questions included how many cigarettes the participant had smoked in the previous week, whether smoking cessation aids (ie, nicotine replacement therapy) had been used, and a self-rating of how stress was perceived. Responses to psychosocial measures were recorded by the study coordinator.

#### Interviews

At the end of pilot 1, 14 participants participated in semistructured telephone interviews regarding their experiences with the SmokeBeat program with research personnel (KS and CMJ). Interviews were focused on which aspects of the program assisted with their smoking cessation attempt and included open-ended questions for participants to talk about the aspects they found most beneficial. Verbal informed consent was obtained before the start of the interview, and audio recordings of every interview were transcribed verbatim by a third-party transcription firm. Participants were paid for participation in the interviews.

#### Cotinine Testing (Saliva+Urine)

A saliva or urine cotinine test was completed during each weekly remote study visit. The type of test was alternated, with participants completing four urine cotinine tests and eight saliva cotinine tests. Saliva cotinine results were obtained using the Alere iScreen Cotinine Oral Fluid Screening Device (Abbott Pharmaceuticals). Participants self-administered these tests during the video chats with a study coordinator. Tests were initiated on a camera with the assigned staff member to ensure that new, unused saliva tests were being used. Participants showed the results panel to the study coordinator via the phone-based camera for the study coordinator to read and record.

Urine cotinine samples were prepared by participants at home. The study coordinator guided the participants by labeling a sample cup, and then waited on the video chat while a sample was obtained. In a few cases, participants prepared urine samples immediately before the video call. Using the labels sent to them, the participants packaged the samples for a UPS (United Parcel Service) pickup. The study coordinator then contacted UPS to schedule the pickup. All urine samples were sent to ARUP Laboratories (Salt Lake City) for analysis. The test results were sent to the study coordinator.

#### Remote Monitoring Data

SmokeBeat uses data from a smartwatch gyroscope and accelerometer plus a proprietary machine learning algorithm to detect smoking episodes from hand and arm gestures. When paired with a smartphone app, SmokeBeat forwards smoking data to a dashboard that can be accessed by a clinician or researcher and delivers context-sensitive messaging to watch wearers about the timing, frequency, and location of smoking. The study coordinator obtained watch-wearing and smoking data directly from the provider or researcher dashboard or from daily summary emails from Somatix staff.

### Outcome Measures, Sample Size, and Analysis

Pilot 1 was calculated for the minimum sample size needed for 70% power to detect a difference of 6 cigarettes in the primary outcome (change in the self-reported cigarettes smoked per week from the beginning to the end of the study) in an unbalanced sample of 30 participants. Pilots 2 and 3 were not powered. Secondary outcomes included cotinine testing results, self-reported psychosocial measures, watch wearing, and app-detected cigarettes. Descriptive statistics were generated from the questionnaire and the SmokeBeat dashboard data. The primary outcome was compared across the treatment and control groups in pilots 1 and 2 using covariate analysis. Secondary outcomes were examined using Poisson regression and two-sided *t* tests. Feasibility and acceptability were assessed through recruitment and retention measures, smartwatch and app linkage and functioning, incentive calculation and delivery, and the ability to conduct weekly video check-ins and cotinine testing. Quantitative analyses were conducted using RStudio, version 1.2.1335.

Interviews were qualitatively analyzed using a rapid analytic framework [22,23]. A thematic codebook was created by using an a priori coding schema developed by the coders reading a subsample of the interview transcripts. A total of 3 investigators (CMJ, AMB, and CB) independently read and coded two interview transcripts to identify major themes and content codes. The investigators independently coded the themes and then created the codebook via discussion until a consensus was reached. This preliminary codebook was used by the investigators in an additional interview to confirm that agreement was reached. Once the codebook was finalized, 3 investigators (SM, CMJ, and JS) coded the rest of the interviews using NVivo 11 (QSR International Pty Ltd). One investigator (CMJ) synthesized the coded interviews into the main themes via the rapid analytic framework.

### Human Subjects Approval and Trial Registry

All pilot studies were approved by the University of Pennsylvania Institutional Review Board and registered with ClinicalTrials.gov (NCT03209557).

## Results

### Pilot 1

#### Participants

Of the 106 eligible pregnant women, 27 (25.5%) consented to participate in the survey. Reasons for refusal to participate in the study varied but centered around a lack of interest in the study. Among the 27 women who consented to the study and were randomized, 25 (93%) completed the baseline demographic survey and 21 (78%) completed the week 1 interview. Of the 21 participants, 14 (67%) were in the intervention group and 7 (33%) were in the control group. A total of 16 participants completed the 12 weeks of the study (Multimedia Appendix 2). The sociodemographic information of the 25 participants who completed the pilot baseline survey is presented in Table 2**.** All women were pregnant at the time of recruitment, with some giving birth during the study period.

**Table 2:** Pilot 1: sociodemographic characteristics of participants (n=25).

Characteristics	Values
Age (years), mean (SD)	27.3 (4.7)
Married or partnered, n (%)	9 (36)
Completed high school, n (%)	20 (80)
Household income <US $20,000, n (%)	17 (68)
Hispanic, n (%)	6 (25)
Black, n (%)	4 (16)
Unemployed, n (%)	16 (64)
Any previous pregnancy, n (%)	16 (64)
Cigarettes smoked per day, mean (SD)	13.2 (13.8)
US $ per week spent on cigarettes, mean (SD)	30.36 (20.22)
Years of smoking, mean (SD)	11.7 (5.9)
Age at smoking initiation (years), mean (SD)	15 (3.02)
Ever attempted to quit, n (%)	21 (84)
Number of quit attempts, mean (SD)	1.9 (1.5)
Other smokers in household, n (%)	16 (64)
Any coworkers who smoke, n (%)	9 (36)

#### Change in Self-reported Smoking

**Figure 1:**
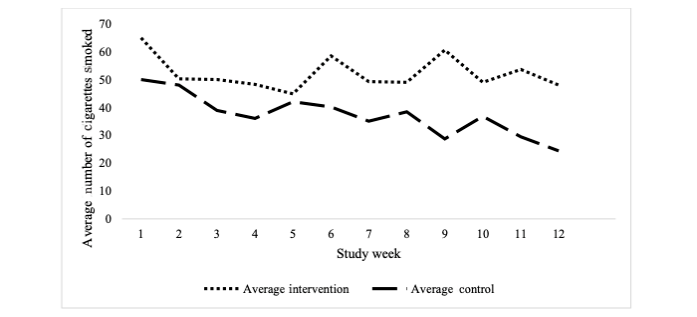
Pilot 1: self-reported cigarettes smoked per week by group.

Self-reported cigarettes smoked per week declined by 15.1 (SD 26) cigarettes in the intervention group and by 17.2 (SD 19) cigarettes in the control group. Among the 21 participants who completed the week 1 and week 12 interviews, the analysis of covariates revealed no significant effect of the intervention on changes in cigarette smoking (*F*_1,14_=0.37; *P*=.55; Figure 1 and Table 3).

**Table 3:** Details of the pilot results.

Pilot	Change in cigarettes smoked per week	Nicotine tests	App engagement	Incentives earned
1	Self-reported cigarettes per week among the intervention group decreased by 15.1 (SD 26) cigarettes; a similar reduction was seen in the control group with a decrease of 17.2 (SD 19) cigarettes.	A total of 6 control participants and 10 intervention participants completed saliva nicotine testing in the final week. In total, 1 out of 6 in the control condition had a negative cotinine test at week 12. Also, 1 out of 10 in the intervention had a negative cotinine test at week 12.	Participants wore the SmokeBeat watch for an average of 57.6 hours total (range: 6-215) on 15.3 different days (range: 2.0-57.0) during the 12-week (84 day) period. Across all participants, the watch was worn for any amount of time on an average of 18% (range: 2.4%-67.8%) of the days in the study period.	Participants were not offered incentives.
2	Self-reported cigarettes per week among the pay-to-wear group decreased by an average of 43 cigarettes (SD 12.6); a smaller reduction was seen in the pay-to-quit group with an average decrease of 31 (SD 45.6).	Intent-to-treat results showed four positive cotinine tests in each condition at the end of the pilot.	Across all participants in the pay-to-wear arm, the watch was worn for any amount of time on 38% (range 10%-75%) of the days in the study period. Across all participants in the pay-to-quit arm, the watch was worn for any amount of time on 77% (range 42%-100%) of the days during the study period.	A total of US $2 were given as incentives—1 dollar per each arm of the study.
3	Participant who completed the study was in the pay-to-quit arm and abstained from smoking during the entire study period.	Owing to NRT^a^ use throughout the study, this participant did not have a negative cotinine test at any study visit.	The participant wore the smartband for the entirety of the study period.	The participant earned a maximum of US $525 incentive payment because of consistent smartband wearing and abstaining from smoking.

^a^NRT: nicotine replacement therapy.

#### Cotinine Testing

**Figure 2:**
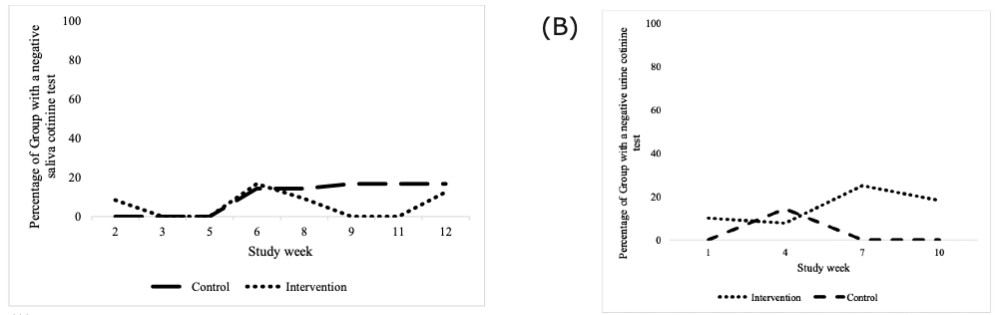
Pilot 1: percentage of tested participants with a negative saliva (A) and urine (B) cotinine test by week and intervention group (intervention maximum: N=14, control maximum: N=7).

Pilot 1 cotinine test results (saliva and urine) are shown in Figure 2. Participants in the intervention group did not have a significantly higher number of negative cotinine tests at final testing compared with the control group. A Poisson regression showed no significant difference in the number of negative cotinine tests at the study end between the arms (incidence rate ratio=1.05; *P*=.93; Table 3).

#### Other Self-reported Measures of Cessation, Craving, Support, and Stress

A total of 5 participants reported using a cessation aid (nicotine patch, nicotine gum, and/or electronic cigarette) at some point during the study; however, none reported consistent use throughout the 12-week period. Participants reported moderate craving or withdrawal (5 on a 1-10 scale) at the time of the interview in week 1, which reduced slightly to 4 by week 12. Participants reported moderate social support (6 on a scale of 1-10) at week 1, which increased to 8 at week 12. The average self-reported level of stress, measured from 1 (no stress at all) to 10 (constant stress), was high in week 1 (8) and moderate in week 12 (5; Table 3**)**.

#### Smartwatch Wearing

In total, 71% (10/14) of the participants in the intervention group had analyzable data on the smartwatch worn by them (Table 3). In general, participants reported issues with their watch battery life and frequently stated that the watch was not holding a charge long enough for it to pick up cigarette use. When appropriate, watches and charging cables were replaced. Participants were given instructions to charge the watch overnight to maximize their battery life.

#### App-Reported Smoking

Participants manually entered an average of 66.5 (0.79/day) cigarettes into the SmokeBeat app while in the study, and the watch detected an additional 17.3 (0.2/day) cigarettes. Combined with the watch-wearing data above, this translates into 0.3 cigarettes detected by the watch per hour watch worn.

#### Interviews

In total, 67% (14/21) of the participants participated in the qualitative interviews. Four major themes emerged from qualitative interviews (Table 4). Participants said that the questionnaires were helpful in realizing that their stress levels contributed to how much they were smoking. They also mentioned that weekly check-ins were helpful as both motivation and social support. Finally, for future studies, they recommended targeted text messages and financial incentives as the most salient motivators for quitting smoking. This information was incorporated into the study protocols for the subsequent pilots.

**Table 4:** Results from qualitative interviews in pilot 1.

Themes	Quotes
Stress	“The questionnaires actually helped to actually think of stress level and how much of it I had.”
Financial incentives	“[if]...you’re [smoking less]. I'm going to pay you...[but if you smoke] then I'm going to pay you a lot less...that’s going to encourage me to want to quit smoking and get that higher amount of money.”
Tailored messaging	“Even just a message daily that, oh you’re down two cigarettes would be, I think, enough of a reward without like a financial reward or prizes.”
Social support through video chat	“Having a motivator, that would be very helpful because I would have someone to talk to and to relate to.”

### Pilot 2

#### Participants

All participants from pilot 1, along with eligible women recently enrolled in the pregnancy support programs, were contacted about participating in pilot 2. In total, 75% (6/8) of the participants were in the pilot 1 intervention group, leaving 25% (2/8) who were naïve to SmokeBeat at the start of pilot 2. All pilot 2 participants were postpartum. A total of 88% (7/8) of participants completed all 4 weeks of the study (Multimedia Appendix 3).

#### Change in Self-reported Smoking

**Figure 3:**
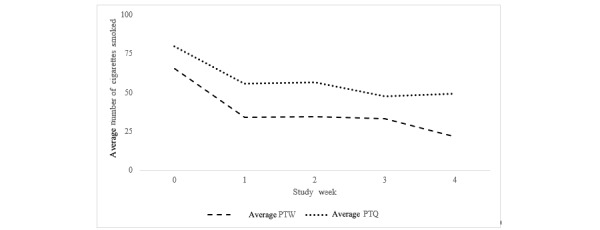
Pilot 2: self-reported cigarettes smoked per week by intervention group. PTQ: pay-to-quit; PTW: pay-to-wear.

At baseline, the average number of cigarettes smoked in the previous week was 72.7 (SD 47.9). The average number of cigarettes smoked the week before decreased to 35.4 (SD 31.6) cigarettes in week 4 **(**Figure 3 and Table 3**).** An analysis of covariates revealed no main effect of intervention on change in cigarettes smoked between week 0 and week 4 of the study (*F*_1,4_=0.64; *P*=.47).

#### Cotinine Testing

Of the 7 participants who completed cotinine testing at week 4, 3 (43%) were in the pay-to-wear condition and 4 (57%) in the pay-to-quit arm. There were no negative cotinine tests performed at week 4. There were no significant differences in negative cotinine test results between the groups (Table 3).

#### Other Self-reported Measures of Cessation, Craving, Support, and Stress

In total, 43% (3/7) of the participants reported using a cessation aid (medication, electronic cigarette, or both) throughout the study. Participants reported moderate craving or withdrawal at the time of the interview in week 0 (6 on a scale of 1-10), which reduced slightly to 3.5 by week 4. Participants reported moderate social support (6 on a scale of 1-10) at week 0, increasing to 8 at week 12. The self-reported stress levels (measured from 1 [no stress at all] to 10 [constant stress]) was 8 in week 0 and 5 in week 12.

#### Smartwatch Wearing

**Figure 4:**
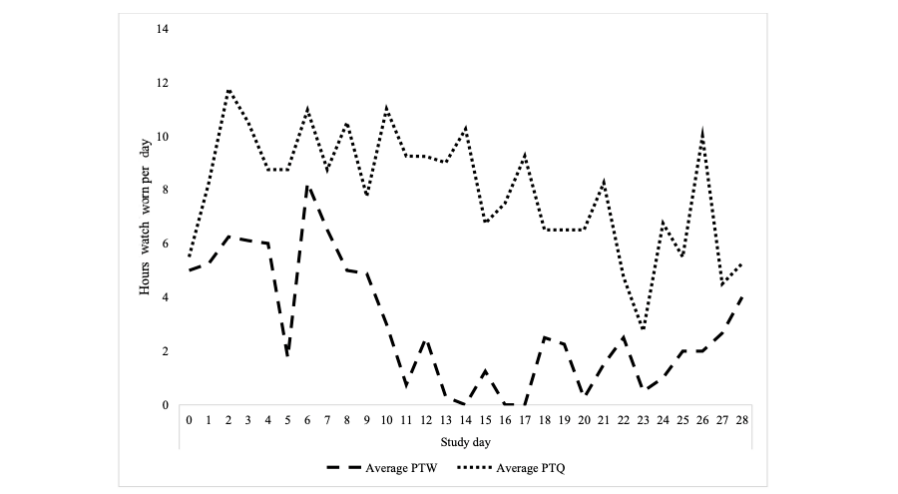
Pilot 2: average number of hours watch worn for each study day by arm (includes days with no watch wearing). PTQ: pay-to-quit; PTW: pay-to-wear.

Participants in the pay-to-quit arm group wore their watches more hours per day than the pay-to-wear arm group (Figure 4), and on more days (Table 3). A two-sample *t* test revealed a significant difference in average watch wearing per day by group (t_57_=−8.6; *P*<.001). Participants reported various reasons for not wearing the watch, including being unable to do so while working and not wanting to wear it around children.

#### App-Reported Smoking (Watch Detected and Manual)

On average, the smartwatch picked up 13 (0.5 per day) cigarettes over the course of the 4-week study for the pay-to-wear group and 55 cigarettes for the pay-to-quit group. Participants in the pay-to-wear group manually entering an average of 3.0 (2.0 per day) cigarettes. Combined with the watch-wearing data above, this translates into 0.05 cigarettes detected by the watch per hour watch worn in the pay-to-wear group and 0.1 cigarettes detected by the watch per hour watch worn in the pay-to-quit group. Participants used the app to input cigarettes even when they did not wear the watch. Participants in the pay-to-quit group entered an average of 1.0, whereas participants in the pay-to-quit group entered on average of 9.0.

#### Incentives Earned

Participants in the pay-to-wear arm were eligible to receive US $1 for every day in which they wore the smartwatch for ≥16 hours. Throughout the study’s duration, 1 participant earned an incentive for 1 day of wearing the watch. Similarly, the pay-to-quit arm had 1 participant who on one day both wore the watch for more than 16 hours and smoked zero cigarettes that day. Overall, only US $2 of incentives were earned during pilot 2.

### Pilot 3

One pregnant pay-to-quit participant completed the 3-week study and abstained from smoking for the entire course of the study following a positive cotinine test at baseline, earning the maximum incentives and bonuses (US $525; Table 3**)**. Following the study protocol (Table 1), incentives were paid based on wearing the watch for >16 hours per day and having the watch record no smoking events during that time. The other 3 participants (1 pay-to-quit and 2 pay-to-wear) were recruited, consented, randomized, and received a smartwatch, but despite multiple contact attempts, no study visits were completed and no watch-wearing or smoking data were recorded via the app (Multimedia Appendix 4). Of these participants, there was some watch wearing in the pay-to-wear group. One participant wore her watch for any amount of time on 13 days, on 4 of which the watch was worn for at least 16 hours; the other wore the watch for one day before ceasing use. The second participant in the pay-to-quit group recorded no watch wearing.

## Discussion

### Principal Findings

We conducted three rapid cycle pilots of remote participant monitoring and incentives to support smoking cessation among pregnant and postpartum Medicaid members. The pilots were designed to maximize learning about the feasibility and acceptability of the intervention components, to assess effectiveness, and to establish study protocols for future randomized trials. Pilot 3, which offered the highest financial incentive amount, had 1 participant in the pay-to-quit condition abstain from smoking for the entire study period. In pilots 1 and 2, cigarettes smoked per week decreased more in the intervention group than in the control group (though not significantly so), suggesting that a scaled-up version of this program may be effective in this population. This is consistent with previous research showing financial incentives to be an effective mechanism for smoking cessation [10], even in harder-to-reach populations [11].

The first goal of the pilot was to establish the feasibility and acceptability of recruiting and conducting a technology-supported, incentive-based cessation program for Medicaid-eligible pregnant smokers. Recruitment through existing prenatal care support programs proved feasible and acceptable to both clinicians and patients, and we were able to recruit 25.5% (27/106) of eligible participants who were approached regarding the study (Multimedia Appendix 2). The feasibility and acceptability of the Somatix SmokeBeat program was fair. Low participant engagement with SmokeBeat in pilot 1 was driven by the short battery life of the smartwatch, with an increased engagement in pilots 2 and 3 when a watch with a longer battery life was provided. Qualitative interviews conducted at the end of the pilot indicated interest in further iterations of the program. Recommendations from these interviews were incorporated into the design of subsequent pilots.

The second goal of the pilot study was to evaluate the role of SmokeBeat in remotely tracking smoking behavior and providing feedback to participants. SmokeBeat provided real-time, passive monitoring and tracking of smoking behavior. Participants who actively used the watch received data via a personalized dashboard about temporal patterns of smoking, progress toward reducing or quitting smoking, and messages targeting social, emotional, financial, and rational motivations to quit. In contrast to some previous smoking cessation research, we did not screen participants for motivation or readiness to quit [24]. It may be the case that the tracking and feedback functions of a program like SmokeBeat are most effective for those already planning to quit. Although most of our pilot participants did not regularly engage with the SmokeBeat app, this type of tailored, real-time feedback to support incentive-based cessation efforts is worth further investigation.

Our third goal was to assess the feasibility and acceptability of conducting remote cotinine testing with participants via video chat for the biochemical verification of smoking status. Remote verification is crucial to scale incentive-based programs and decouple them from burdensome clinic visit schedules. We established the feasibility and strong acceptability of receiving cotinine testing supplies by mail and conducting the testing live via video chat with a study coordinator. Participants who could not provide a urine sample at the time of the video check-in, or who did not fully saturate the saliva test (resulting in an incomplete test result), were able to complete these steps at a later time and notify the study coordinator. Although busy schedules during pregnancy and the postpartum period also meant that video check-in appointments were frequently missed or rescheduled, overall, we observed a very good engagement with the check-ins over the duration of the pilots.

Cotinine testing was generally consistent with RPM data from self-reported tobacco use and SmokeBeat, except when a participant had stopped smoking cigarettes but was using nicotine replacement therapy, which results in a positive cotinine test. In these cases, biochemical verification would report positive cotinine results, whereas the dashboard would show zero smoked cigarettes.

Finally, we hoped to establish a feasible and acceptable protocol for delivering the incentives of different magnitudes contingent on smoking cessation or engagement with remote tracking technology. Our results are clearly positive and promising for future studies. Earned incentives were straightforward to calculate from the SmokeBeat dashboard and data sent by Somatix, and incentives were easily and immediately loaded onto ClinCards. Participants had minimal issues using ClinCards for routine daily purchases, including groceries and gas.

The limitations of this series of pilots include overall low patient volumes from which to recruit, challenges with smartwatch battery life that limited the ability to engage with the SmokeBeat platform, and a substantial variation in motivation to quit. These limitations, along with variability in working hours and family commitments, likely contributed to attrition from the study as many participants had to prioritize other activities over study participation. In addition, both pregnant and recently postpartum women participated in these pilots, and it is possible that differences between these groups led to differential smoking behavior. There is also the possibility of a social desirability effect from this research study, as both control and intervention participants received weekly check-in calls with study staff. This could have led to bias in the self-reported cigarette outcome and contributed to the overall decrease in smoking observed in the intervention and control groups in pilots 1 and 2. Broader limitations to an incentive-based program with a similar design to our pilots include the persistent structural and environmental barriers to successful tobacco cessation faced by our target population of Medicaid-eligible pregnant women. Daily stressors related to resource scarcity, employment challenges, and transportation insecurity, for example, may make smoking an important source of relaxation, stress relief, and pleasure and indulgence that women are reluctant to give up. Living in households with other smokers can compromise or undermine quit attempts. The financial incentive payments used in this study were modeled after previous smoking cessation studies [10,21], and created in conjunction with the employees of a hospital-based program aimed at Medicaid-eligible pregnant women to ensure they did not reach the level of coercion. However, with any financial incentive study, there is a possibility that participants enrolled because of financial incentives alone. To mitigate this, participants were paid for survey completion to ensure that their remuneration did not rest solely on smoking cessation. Effective, scalable solutions to support tobacco cessation in this population must consider the social and economic contexts and the cultural and emotional benefits of smoking.

### Conclusions

Our pilot study demonstrates the feasibility and acceptability of several crucial operational components of a technology-supported, incentive-based smoking cessation program for Medicaid-eligible pregnant women that leverages RPM. We remain optimistic about the potential for incentives to boost existing tobacco control programs through scalable and sustainable innovations.

### Declarations

#### Ethics Approval and Consent to Participate

This study was approved by the University of Pennsylvania Institutional Review Board (protocol #827096).

#### Consent for Publication

Not applicable.

#### Availability of Data and Materials

The data sets used and/or analyzed during this study are available from the corresponding author upon reasonable request.

## Appendix

Multimedia Appendix 1 Weekly survey questions.

Multimedia Appendix 2 CONSORT (Consolidated Standards of Reporting Trials) diagram for pilot 1.

Multimedia Appendix 3 CONSORT (Consolidated Standards of Reporting Trials) diagram for pilot 2.

Multimedia Appendix 4 CONSORT (Consolidated Standards of Reporting Trials) diagram for pilot 3.

Multimedia Appendix 5 CONSORT-eHEALTH checklist (V 1.6.1).

## Abbreviations

RPM: remote patient monitoring
UPS: United Parcel Service
