# Supplementary material for: Remote Patient Monitoring and Incentives to Support Smoking Cessation Among Pregnant and Postpartum Medicaid Members: Three Randomized Controlled Pilot Studies
Source: JMIR Form Res. 2021 Sep 30;5(9):e27801. doi: 10.2196/27801 (PMC8517817; doi:10.2196/27801)
Supplement: Multimedia Appendix 1 [file formative_v5i9e27801_app1.docx]

**Multimedia Appendix 1.** Weekly survey questions.

1. How many cigarettes did you smoke last week?
2. Have you used any cessation aids this week, if so which ones (NRT, e-cigarettes)?
3. On a scale of 1-10, with 1 being “I don’t need a cigarette at all” and 10 being “I really need a cigarette”, how strong is your craving/withdrawal right now?
4. On a scale of 1-10, with 1 being “I don’t need a cigarette at all” and 10 being “I really need a cigarette”, how strong is your craving/withdrawal this week?
5. On a scale of 1-10, with 1 being “no social support” and 10 being “all the support I need”, how much social support do you have this week this week?
6. On a scale of 1-10, with 1 being “no stress at all” and 10 being “constant stress”, how much stress have you experienced this week?
