# Supplementary figures and images for: Remote Patient Monitoring and Incentives to Support Smoking Cessation Among Pregnant and Postpartum Medicaid Members: Three Randomized Controlled Pilot Studies
Source: JMIR Form Res. 2021 Sep 30;5(9):e27801. doi: 10.2196/27801 (PMC8517817; doi:10.2196/27801)

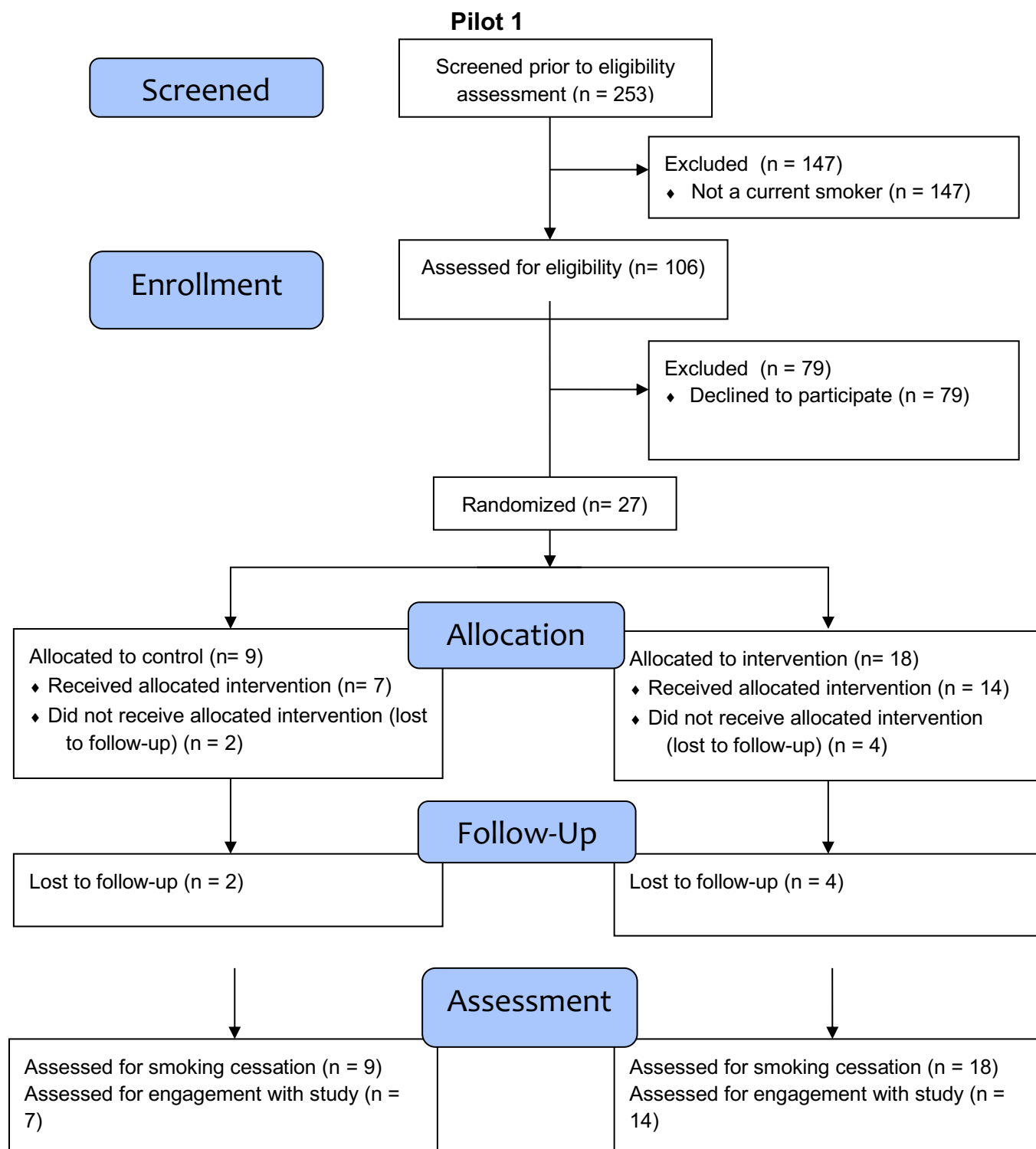

Supplementary Figure 1. Consort Diagram

Supplement: Multimedia Appendix 2 [file formative_v5i9e27801_app2.pdf]

## Pilot 2

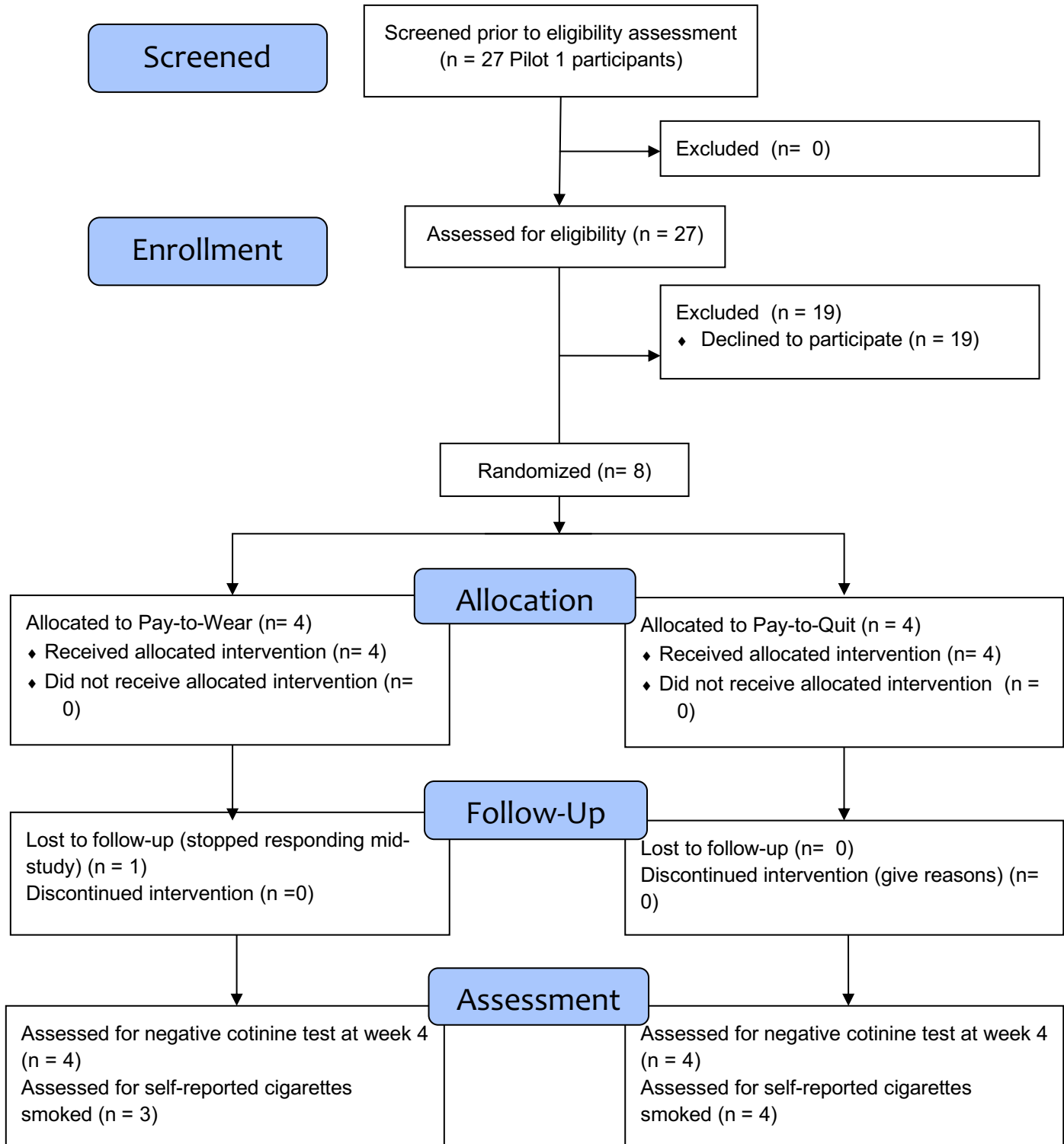

**Supplementary Figure 2.** Consort diagram for pilot 2

Supplement: Multimedia Appendix 3 [file formative_v5i9e27801_app3.pdf]

### Pilot 3

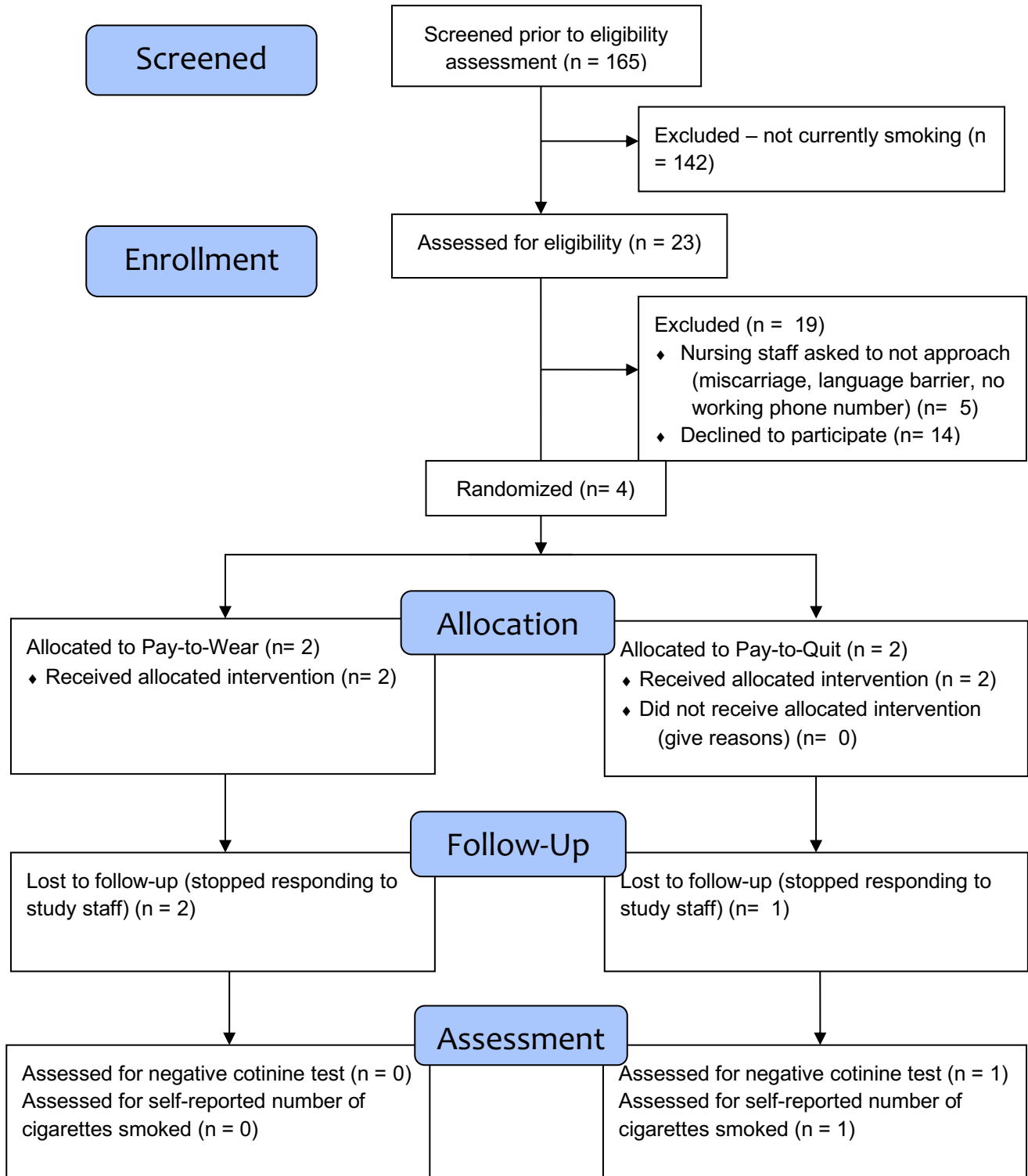

**Supplementary Figure 3.** Consort diagram for pilot 3

Supplement: Multimedia Appendix 4 [file formative_v5i9e27801_app4.pdf]
